# Self-assembly of glycerol monooleate with the antimicrobial peptide LL-37: a molecular dynamics study†

**DOI:** 10.1039/c9ra10037g

**Published:** 2020-02-26

**Authors:** R. Innocenti Malini, M. Zabara, M. Gontsarik, K. Maniura-Weber, R. M. Rossi, F. Spano, S. Salentinig

**Affiliations:** Empa, Swiss Federal Laboratories for Materials Science and Technology, Laboratory for Biomimetic Membranes and Textiles Lerchenfeldstrasse 5 9014 St. Gallen Switzerland riccardo.innocentimalini@empa.ch; Laboratory for Biointerfaces, Department Materials meet Life Lerchenfeldstrasse 5 9014 St. Gallen Switzerland; Department of Chemistry, University of Fribourg Chemin du Musée 9 1700 Fribourg Switzerland stefan.salentinig@unifr.ch

## Abstract

Over the past decade, the rapid increase in the incidence of antibiotic-resistant bacteria has promoted research towards alternative therapeutics such as antimicrobial peptides (AMPs), but their biodegradability limits their application. Encapsulation into nanocarriers based on the self-assembly of surfactant-like lipids is emerging as a promising strategy for the improvement of AMPs' stability and their protection against degradation when in biological media. An in-depth understanding of the interactions between the structure-forming lipids and AMPs is required for the design of nanocarriers. This *in silico* study, demonstrates the self-assembly of the amphiphilic lipid glycerol monooleate (GMO) with the antimicrobial peptide LL-37 into nanocarriers on the molecular scale. Molecular dynamics (MD) simulations show the formation of direct micelles, with either one or two interacting LL-37, and vesicles in this two-component system in agreement with experimental results from small-angle X-ray scattering studies. The hydrophobic contacts between LL-37 and GMOs in water appear responsible for the formation of these nanoparticles. The results also suggest that the enhanced antimicrobial efficiency of LL-37 in these nanocarriers that was previously observed experimentally can be explained by the availability of its side chains with charged amino acids, an increase of the electrostatic interaction and a decrease of the peptide's conformational entropy upon interacting with GMO. The results of this study contribute to the fundamental understanding of lipid–AMP interactions and may guide the comprehensive design of lipid-based self-assembled nanocarriers for antimicrobial peptides.

## Abstract 2

Molecular dynamics simulations of glycerol-monooleate (GMO)/LL-37 nanocarriers show that hydrophobic interactions among the molecules drive the formation of GMO/LL-37 micelles.

## Graphical abstract

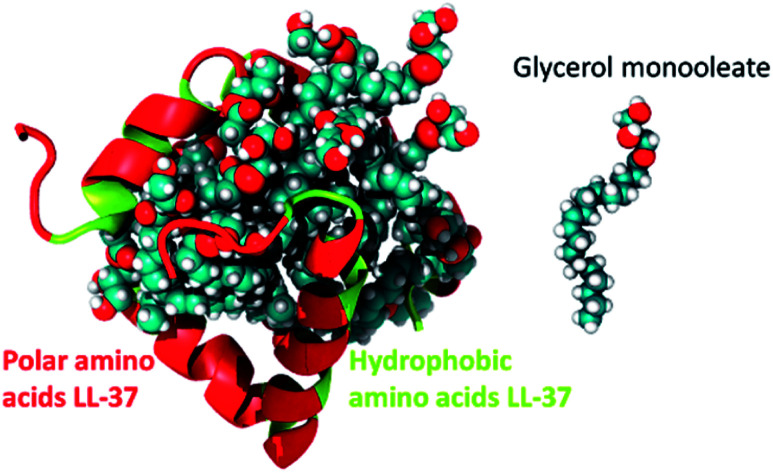

## Introduction

Antimicrobial peptides (AMPs) have gained increasing attention as a promising alternative to conventional antibiotics due to their unspecific mode of action and their broad range of activity.^1–4^ However, their limited stability in biological media due to degradation remains a significant challenge for their pharmaceutical application.^5–9^

LL-37 is a cathelicidin-derived peptide found in humans that is pivotal for many biological functions. It displays broad antimicrobial activity against Gram-positive and negative species,^10–12^ it can modulate the immune cell response to sites that are infected,^11,13^ and it affects the inflammatory reactions of the body.^14,15^ LL-37 is composed of 37 residues (listed in Fig. S1†), of which 43% are charged, 43% are hydrophobic, and 14% are polar. Of the charged amino acids, 69% are basic, entailing that at a pH of 7, LL-37 carries a net positive charge of 6, an important factor in its antimicrobial activity because of electrostatic attractions to the generally negatively charged microbial cell walls.^16^ Another critical aspect of its antimicrobial activity is its secondary structure. LL-37 forms an amphipathic helical structure when in salt solutions or contact with lipid membranes.^17^ Separation of the hydrophobic and hydrophilic groups into two different regions forces LL-37 to either oligomerize with other LL-37 peptides, a process that has recently been proposed as an initial step to destabilize membranes or to interact with the hydrophobic aliphatic chains of lipid membranes.^18,19^ Once the concentration of the peptide on the microbe membranes reaches a threshold level, it starts to destabilize it, eventually leading to the pathogen's death.^19–21^

Pathogens have evolved defense mechanisms against antimicrobial peptides, including the production of proteinases that can cleave LL-37 and either inhibit his activity or severely hamper its action.^22^ Sieprawska-Lupa, for instance, showed that *Staphylococcus auresus's aureolysin* (a metalloproteinase) was able to cleave and inactivate LL-37 and that *Staphylococcus aureus* strains with higher expression of metalloprotease were less susceptible to LL-37 activity.^7^ In another investigation, Thwaite *et al.* investigated the susceptibility of *Bacillus species* to LL-37, again observing that the species that secreted metalloproteases showed the highest resistance to LL-37.^23^

Nanocarriers based on the self-assembly of surfactant-like lipids are considered as a potential solution for the encapsulation and delivery of AMPs. They can solubilize hydrophilic, hydrophobic, and amphiphilic molecules and protect them from degradation while improving their bioavailability and efficiency.^24–31^ In particular, nanocarriers based on glycerol monooleate (GMO) have attracted significant interest due to the ability of this amphiphilic lipid to form an inverse bicontinuous cubic phase in excess water that can provide large lipid-water interfacial areas for the encapsulation of bioactive molecules.^26,32–35^

Previous investigations have demonstrated that the antimicrobial peptide LL-37 could be loaded into dispersed GMO liquid crystalline nanoparticles with an internal inverse bicontinuous cubic phase of *Im*3*m* and *Pn*3*m*-type symmetries.^24–26^ Small-angle X-ray scattering (SAXS) analysis of the nanostructures showed that the addition of low concentrations of LL-37 (≤5 wt% relative to GMO) to the GMO dispersions increased the dimensions of the GMO's cubic lattice and that, upon reaching concentrations above 5 wt%, LL-37 favored the formation of sponge-like structures and vesicles.^26,36^ Further increase in the LL-37 content in the self-assemblies to a GMO/LL-37 weight ratio of 1 : 1 led to the formation of vesicles and micelles.^26^ These GMO/LL-37 nanocarriers displayed enhanced activity *in vitro* against a clinically relevant *Escherichia coli* strain when compared to free LL-37.^26^ It was discussed that the GMO/LL-37 self-assemblies might act as a shuttle for LL-37, facilitating the accumulation of higher local peptide concentration at the bacteria membrane.^26^

The increased antimicrobial activity could also be associated with a change in the peptide's conformation upon interaction with the GMO molecules. Indeed, it is well known that ions,^17^ lipids,^37–40^ nanoparticles,^41^ and other small molecules like suramin^42^ or ibuprofen,^43^ can affect the structure and function of antimicrobial peptides. In this context, improvements in wound healing activity of gold nanoparticles (Au–NP)/LL-37 complexes *in vivo* and *in vitro* over LL-37 in solution, was also rationalized by a change in the conformation of the peptide upon adsorption.^41,44^ Molecular dynamics (MD) simulations demonstrated that the peptide had a larger radius of gyration and solvent accessible surface area on the Au–NP than in solution. Additionally, the positively charged amino acids of LL-37 were found to remain in the solution when LL-37 was bound to the Au–NP.^41^ The higher availability of these functional groups and their arrangement were therefore used to explain the enhanced activity of the composite.^41^ In another study, 2D and 3D-NMR have been used to analyze the conformation of LL-37 when bound to model micelles, namely sodium dodecyl sulfate (SDS), dioctanoylphosphatidylglycerol (D8PG) and dodecylphosphocholine (DPC).^38,39^ In these systems, LL-37 was found to adopt an α-helical conformation in its central region, forming an amphipathic structure where the hydrophobic amino acids anchored the peptide to the micelles. Instead, the C-terminal was always found in a random coil conformation and able to arrange into various conformations.^38,39^ The N-terminal conformation, however, depended on the molecule chemistry. On DPC, it formed a random coil secondary structure that was mobile, while on SDS and D8PG it adopted an α-helical conformation and was rigid.^38,39^

The nanostructure of the antimicrobial GMO/LL-37 self-assemblies and the conformation of the peptide in these systems are considered to be crucial factors for the biological activity of the AMP-loaded nanocarriers.^17^ However, the detailed mechanisms underlying the self-assembly of GMO and LL-37 and its impacts on the conformation of LL-37 is mostly unknown. Towards this goal, MD simulations were used to research the molecular interactions between GMO and LL-37 guiding their self-assembly into nanostructures, and the conformation of LL-37 in these structures. The simulations show the formation of micelles and vesicles in the GMO/LL-37 mixture at a 1/1 weight ratio. LL-37 was found to stabilize the micelles through hydrophobic contacts, while the polar residues were found to remain in solution. Furthermore, the entropy of the peptide was found to change significantly upon binding to the GMO self-assemblies. Together with the shuttle mechanism in the GMO/LL-37 nanostructures, this change in entropy may also contribute to the previously reported increase in the antimicrobial activity of the LL-37 in this complex in experimental studies.^26,45^

## Methods

### Simulation methods

The LL-37 and GMO (later also denoted as organic molecules) were modeled using the AMBER force field, which is often employed for the simulation of proteins in combination with lipids.^46–48^ More specifically, the GMO's aliphatic tail was modeled using LIPID14, while the generalized amber force field (GAFF) was used for the glycerol head group.^49–51^ For the peptide, the ff14SB model was chosen.^52^ All models are compatible as AMBER force fields are built using a modular approach.^52^ The water was simulated using the SPC/Fw model.^53^ Chloride was added to the system in order to equilibrate the charge.^54^

The benchmarking of the force field used for the GMO (explained in the following section) was done using DL_POLY_classic version 1.9.^55^ Then, to take advantage of GPU accelerated nodes to speed up the calculations, LAMMPS was used to study the LL-37–GMO interactions.^56–59^ In both cases, the production run used the NPT ensemble (constant number of particles, pressure, and temperature; isothermal-isobaric ensemble) to keep the temperature and pressure at 310 K and 1 atm, respectively.^57^ This was achieved by using a Nosé–Hoover thermostat and barostat with 0.1 ps and 1.0 ps relaxation times, respectively.^60^ The cutoff for all the intermolecular interactions was set at 10 Å, and the electrostatic contributions to the potential energy were calculated *via* the particle–particle–particle–mesh method.^61^ The time step for the simulations was 1 fs to ensure that the bonds involving hydrogen bonds remained stable.

Snapshots of the trajectories produced during the MD simulations were obtained by using the VMD software.^62^

### Benchmarking GMO

While AMBER modular philosophy allows mixing force fields, it was important to verify that the GAFF parameters for the glycerol head could be used to model the GMO molecules. Two validations were performed to ensure that the structures obtained were comparable to previous experimental and simulation results.^63^ In the first one, the radius of gyration (*R*_g_) of reverse GMO micelles in toluene was compared to previous simulations that used the OPLS-AA all-atom force field.^63^ The pure toluene simulation density was 841.1 kg m^−3^ and was closer to experimental values compared to the value calculated using the OPLS-AA force field.^63^ As expected, when GMOs were inserted in the box, it formed a reverse micelle, with a *R*_g_ of 16.1 ± 0.5 Å. This is 1.7 Å larger than the previous experimental work (14.4 Å).^63^ To ensure that the interactions with water were also sensible, a GMO bilayer was simulated in contact with water (average system size: 30.9 × 30.9 × 70.2 Å^3^) using the NPT ensemble, with the temperature set at 310 K and the pressure at 1 atm. After 100 ns of simulation, the area per GMO molecule was 0.34 lipids nm^−2^, in good agreement with previous simulations and experiments that found values between 0.32 and 0.36 nm^2^.^64–66^ The electron density of the system is presented in Fig. S2† showing that the overall membrane thickness was 31.9 Å and that the bulk water electron density was 0.33 e Å^3^, which is the usually expected value. Therefore, the chosen model appeared to work well and was used in the subsequent simulations.

### Preparation of GMO/LL-37 simulations

Two systems were prepared; one containing 200 GMO molecules and 100 000 water molecules and a second one with 200 GMO, 16 LL-37, and 100 000 water molecules. The number of LL-37 to GMO was chosen to have a 1 : 1 wt ratio as the corresponding experiments.^45^ Initially, the two simulations were started from a random configuration of the molecules in a cubic box generated using Packmol.^67^ To insert the molecules in the system, the minimum distance (tolerance) between the atoms in the different molecules was set to 2.3 Å. The systems were relaxed in the NVT ensemble (constant number of particles, volume, and temperature; canonical ensemble) for 200 ps using a time step of 1 fs to remove the high strains in the system arising from the random packing process.^60^ Then, the ensemble was changed to NPT (as described above), and the simulations were run for 24 ns.

At the end of these large-scale simulations, the GMO/LL-37 micelles that formed contained between one and two LL-37 apart from one that was composed entirely of GMOs. Of this ensemble, two micelles that formed in the presence of LL-37 were extracted and simulated in a system containing 12 000 water molecules to equilibrate them further. In the simulations, one of the two micelles had one LL-37 interacting with its surface (for subsequent reference, this peptide is labeled as LL-37_MS_) while the other one had two (labeled as LL-37_MT1_ and LL-37_MT2_). The water in the system was initially equilibrated in the NVT ensemble for 200 ps while keeping the organic molecules fixed in their position. After this step, the organic molecules were allowed to move freely, and the ensemble was switched to NPT and simulated for 200 ns. In these simulations, no additional ions were added to the simulation to equilibrate the charge. The ions interactions with the peptide would have required extensive additional simulations to ensure that the different configurations were sampled appropriately, thereby increasing the complexity of the simulations. Instead, the implicit neutralizing background was used. This method introduces a uniform charge density throughout the system. Because the systems contain a hydrophobic region characterized by a lower dielectric constant compared to the polar solution, there is a possibility that this choice could affect the preferred conformation of the peptide, by increasing the interactions of the positively charged groups with the center of the micelles due to the homogeneous distribution of negative charges due to the uniform neutralizing background.^68^ In that case, there should be a trend in the distance between the peptide's functional groups and the center of mass of the micelles, depending on the number of LL-37 presents (one or two LL-37), which in Fig. S3† is not observed. This is probably due to the small size of the hydrophobic region because the micelles are only composed of 15 and 14 GMO molecules leading to a small charge density in this lower dielectric medium.

Additional simulations of LL-37 in water were performed to compare its structure with the LL-37 interacting with the GMO aggregates. In the first simulation, the initial peptide conformation was taken from the simulation of LL-37_MS_. Then, 12 000 water molecules were added to the system, which was simulated in the NPT ensemble for 200 ns (LL-37_α_). For the second simulation, the same system was initially simulated in the NVT ensemble for 7 ns at a temperature of 800 K to denature the secondary structure of the peptide (labeled as LL-37_rand_). The ensemble was then switched to NPT, using a temperature of 310 K and a pressure of 1 atm. The system was simulated for an additional 200 ns. These two systems, LL-37_α_ and LL-37_rand_, were chosen to represent two extremes conformations that LL-37 could display in solution; LL-37 in its alpha-helical structure and in a random coil configuration. These two systems sit in two different local minima that could occur in solution and are used to find the change in conformational entropy of the peptide when interacting with GMOs.

For all simulations, the secondary structure of the peptide was calculated using STRIDE, a program that defines the secondary structure as a function of the energy of the hydrogen bonds, and the backbone torsion angles.^69^ The root mean squared deviation (RMSD) and the conformational entropy were calculated using the GROMACS *trjconv*, *rms*, *covar* and *anaeig* functions.^70^ The *anaeig* function calculates two different entropies, one using the Quasi-harmonic analysis and the other one using the Schlitter's formula.^71^ To compare the results with recent work on LL-37, the conformational entropy obtained from Schlitter's formula is reported.^41^ Statistical significance was investigated using the standard student *t*-test.

### Geometrical analysis of the GMO aggregates

To investigate the effect of LL-37 on the self-assembly of GMO, the gyration tensor of the different clusters was extracted by using the following equation:1
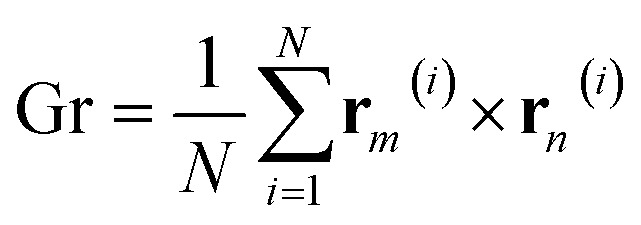
where the superscript *i* indexes the particles' position vector **r**, while the subscripts *m* and *n* index the Cartesian directions. Here, the origin of the position vectors of the atoms composing the molecules was the center of the cluster to which they belonged. The tensors were then diagonalized to extract the principal moments (*λ*_*x*_^2^, *λ*_*y*_^2^ and *λ*_*z*_^2^) that were used to calculate the geometrical properties of the aggregates, including the radius of gyration, *R*_g_ (in Å) and the unit-less relative anisotropy, *κ*^2^:

2 *R*_g_ = *λ*_*x*_^2^ + *λ*_*y*_^2^ + *λ*_*z*_^2^

3
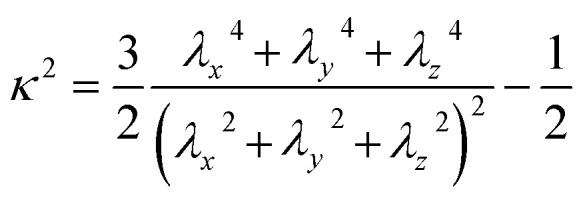


### SAXS profile of the simulated structures

The SAXS pattern of the micelles was obtained by using the Debye equation:^63^4
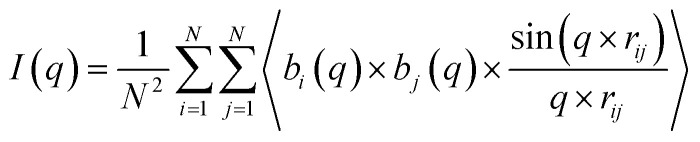
where the reference point was the center of the aggregate. In eqn (4), *I*(*q*) is the scattering intensity as a function of the scattering vector magnitude *q*, *b*_*i*_ represents the scattering length of atom *i*, *q* is the scattering vector, *r* is the distance between the atoms and *N* is the number of particles in the system.

The SAXS profile of the GMO membrane was calculated using SIMtoEXP, a program created by N. Kučerka.^72^

## Results and discussion

### GMO – LL-37 interactions

**Fig. 1:**
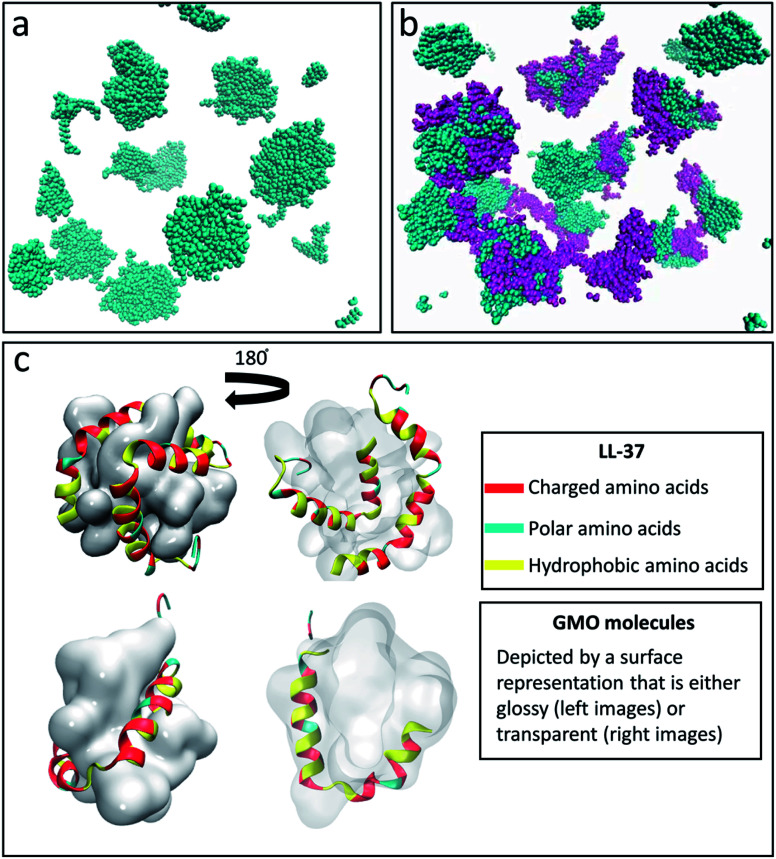
Snapshot obtained after 24 ns of simulation of the system containing (a) 200 GMO and (b) 200 GMO and 16 LL-37 at a 1 : 1 weight ratio. In (a) and (b) the GMO molecules are colored in cyan while the LL-37 is shown in purple. Formation of globular aggregates is observed in both (a) and (b). (c) Front and back view of GMO/LL-37 micelles that were extracted and simulated for 200 ns. In all the images water is not shown for clarity. In (c) the GMO molecules are displayed as a continuous glossy surface and LL-37 using a cartoon representation.

Fig. 1a and b show the final step of the MD simulation concerning the self-assembly of 200 GMO molecules in water after 24 ns in the absence and presence of 16 LL-37 molecules. The latter corresponds to a GMO/LL-37 weight ratio of 1 : 1 that was also used in previous experimental studies.^45^ After a simulation time of 24 ns, the GMO molecules separated from the solvent and formed small aggregates that were used as a reference for the following simulations with GMO and LL-37. The GMO aggregates displayed a surface with hydrophilic (hydroxyl groups from the glycerol head) and hydrophobic (aliphatic chains) domains, see Fig. S4a and b.† Experimentally, GMO was reported to form a reverse bicontinuous cubic phase that coexists with excess water.^73,74^ The large size of this system with the limited simulation time at the computational facility restricted the ability of the GMO molecules to assemble into this thermodynamically favored structure.^74^ The simulation of the GMO's reverse bicontinuous cubic phase in equilibrium, however, was not the aim of this study, which instead focuses on the interactions between the GMO and the LL-37.

**Fig. 2:**
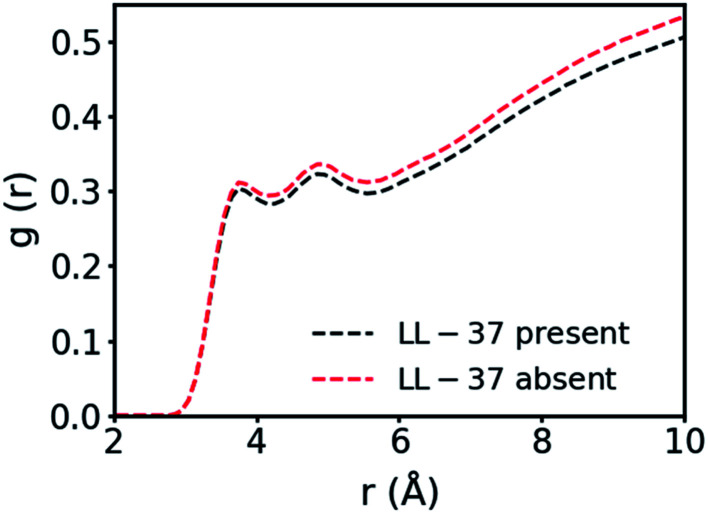
Pair distribution function between the carbon atoms in the GMO's aliphatic chain and the oxygen of the water molecules.

In the simulation containing GMO and LL-37, all the GMO molecules were within aggregates in the form of micelles and all micelles but one contained one or two LL-37 after 24 ns of simulation time (Fig. 1b). To further investigate the interactions in GMO/LL-37 micelles and their structure, two micelles were extracted and simulated for an additional 200 ns in water. These contained either one or two LL-37 and represented the two types of micelles observed in the large scale system. Fig. 1c and S5† show that the LL-37 wraps around the aliphatic domains of the GMO aggregates that are exposed to the solvent, and positions its hydrophobic groups facing those hydrophobic regions. This arrangement reduces the contact between the water molecules and the aliphatic chains of the GMO molecules as well as the hydrophobic amino acids of LL-37 by forming a more continuous polar surface around the self-assembled structure. This leads to the spontaneous formation of direct GMO/LL-37 micelles. The effect of the hydrophobic contacts can be observed in the pair distribution function calculated for the GMO's carbon atoms and the water molecules' oxygen presented in Fig. 2. The curve intensity decreases when the peptide is present, demonstrating that the interaction of the peptide and the GMO aggregates shields the aliphatic chains from the contact with water molecules. An example of these hydrophobic contacts between GMOs and LL-37 is shown in Fig. S5a,† where the phenylalanine aromatic rings are interacting with the GMO aliphatic chains. On the other hand, Fig. 1c illustrates that the polar and charged residues were mostly directed outwards towards the water (red and cyan surface of the peptide – discussed in more detail in the next section). In some cases, however, interactions between charged groups with the glycerol head groups of the GMO *via* hydrogen bonds were also observed. An example of these interactions is shown in Fig. S5b,† where an aspartic acid residue forms a hydrogen bond with the alcohol group present on GMO.

**Fig. 3:**
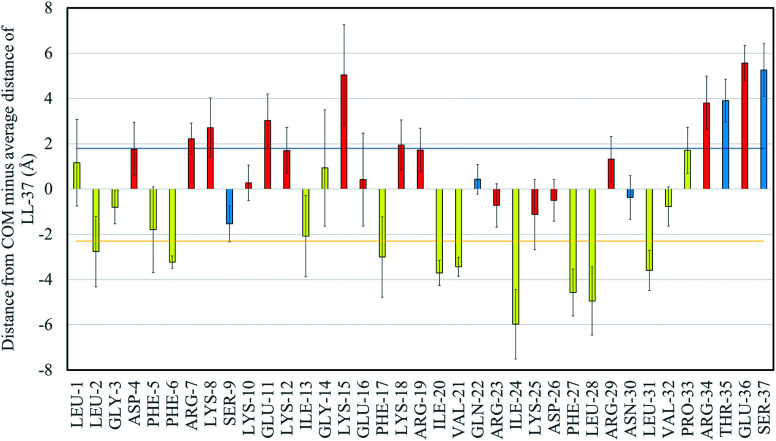
Distances of the center of mass of the different amino acids in LL-37 from the center of mass of the GMO/LL-37 micelles minus the average distance of the peptide. The results presented are an average obtained from LL-37_MS_, LL-37_MT1_, and LL-37_MT2_. Error bars represent the standard deviation of the averages. The dark blue horizontal line represent the average distance of the hydrophilic groups (1.76 Å) while the yellow one is for the hydrophobic groups (−2.30 Å). The charged amino acids are colored in red, the polar ones in blue and the hydrophobic ones in yellow.

The separation of LL-37's amino acids into hydrophobic and hydrophilic regions is observed in Fig. 3, where the distance between the center of mass (COM) of the GMO/LL-37 micelle and the COM of each of the amino acids minus the overall average distance of LL-37 is displayed. Fig. 3 also shows that there are variations between amino acids of the same class (hydrophilic or hydrophobic). This can be explained by considering that the distance to the COM of an amino acid is not only dependent on its functional group, but also on the characteristics of the amino acids that are next to it. For instance, the polar GLN-22, ARG-23, LYS-25, and ASP-26 are in a region that is dense in hydrophobic amino acids deeply embedded in the micelle. This conformation forces these polar amino acids to remain closer to the micelles' surface (Fig. 3). The opposite is true for the hydrophobic amino acids between ARG-7 and GLU-16 that are situated in a region dominated by hydrophilic amino acids and therefore do not penetrate as deep into the GMO aggregates as other hydrophobic amino acids present on LL-37. Other characteristics that are shown in Fig. 3, are the large standard deviations present for some of the amino acids. These variations arise from the different conformations that the functional groups present on the amino acids can adopt. The averages and standard deviations were obtained from the three LL-37 on the two GMO/LL-37 micelles, the first one containing one LL-37 and 15 GMOs and the second one with two LL-37 and 14 GMOs. As shown in the previous section, the hydrophilic groups can interact either with the solution or with the GMO's head group, a difference that will have a substantial impact on the amino acid average position. Additionally, small changes in the LL-37 secondary structure will also affect the average distance of the amino acids.

In general, the hydrophilic amino acids were 4.06 Å further away from the center of the micelle than the hydrophobic amino acids, on average (*p* < 0.05). Of the hydrophobic amino acids, phenylalanine (−3.1 ± 0.3 Å), leucine (−2.5 ± 0.9 Å) and isoleucine (−3.9 ± 0.3 Å) are the ones found closer to the COM of the micelle. This observation is in good qualitative agreement with the previously reported 3D and 2D NMR results for LL-37 on SDS, D8PG, and DPC micelles.^38,39^ In these studies, NOE cross-peaks showed that the aromatic rings of all the phenylalanine were interacting with the D8PG's carbon, present in the head groups and the aliphatic chains of the detergent molecules.^38^ Isoleucine and leucine are also known to be important in anchoring peptides to hydrophobic surfaces.^75,76^ For instance, SMAP-29, an ovine cathelicidin, does not contain any phenylalanine, but instead is rich in isoleucine and leucine, which have been previously shown to be involved in the interaction with lipid structures.^75,76^ The current simulations and previous experimental NMR structural analysis on SDS and D8PG micelles also agree that the LL-37 hydrophobic surface is interrupted by SER-9, a polar amino acid, which in the current simulations is found close to the COM of the GMO/LL-37 micelles, with an average relative distance of −1.53 Å compared to the average distance of LL-37.^38,39^

Of the hydrophilic amino acids, SER-37, GLU-36, THR-35, ARG-34, LYS-18, LYS-15, GLU-11, LYS-8, and ARG-7 are found to be further away than the average distance of the center of mass of the hydrophilic groups, 1.8 ± 0.1 Å. While the difference was significant only for SER-37 and GLU-36, it is still worth to discuss the trend as it could potentially relate to the antimicrobial function of the peptide. The amino acids mentioned above are concentrated in the end regions of the N-terminal, the beginning of the central region (residues 14–31), and the C-terminal region (residues 32–37) of LL-37. The central region is known to be important in the antimicrobial function of LL-37, and exposure of the charged groups toward the solvent when bound on GMO/LL-37 micelles could facilitate interaction with the bacterial cell membrane and its destabilization.^77,78^

### Secondary structure of LL-37

**Fig. 4:**
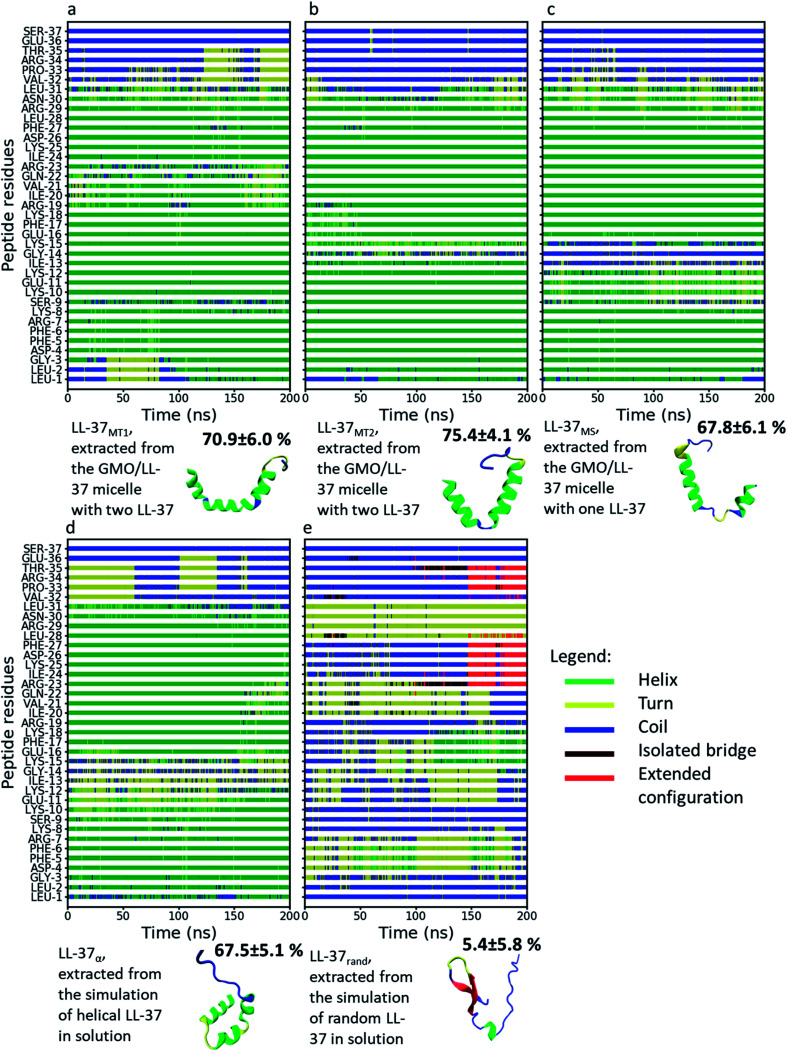
Secondary structure of LL-37 from different MD simulations as a function of time and color-coded representations of LL-37 at the end of these simulations. The colors of the different secondary structures are shown in the legend. The percentage next to each LL-37 snapshot represents the amount of α-helicity in the secondary structure. (a) LL-37_MT1_, (b) LL-37_MT2_, (c) LL-37_MS_, (d) LL-37_α_ and (e) LL-37_rand_.

Fig. 4 displays the secondary structure of LL-37 along its residues as a function of time for all the simulated systems and additionally shows a snapshot of the structures at the end of the simulation. The average percentage α-helicity (calculated using STRIDE^69^ every 2 ps and then averaged throughout the simulations) in the peptide when bound to the GMO/LL-37 micelles (Fig. 3a–c) ranged from 67.8 ± 6.1% (LL-37_MS_) to 75.4 ± 4.1% (LL-37_MT2_), which agrees well with the secondary structure previously obtained from a 3D-NMR analysis of LL-37 in contact with SDS or D8PG micelles (78.4%).^38^ The differences between the simulations and the NMR results obtained for the micelles are associated with the middle region and the terminal region of the peptide (residues 9–15 and 29–31, respectively). In the experiments, these amino acids were labeled as α-helical, while in the current simulations residue 14 is predominantly in a random coil conformation in both LL-37_MS_ and LL-37_MT2_, while the other residues (9–15 and 29–31) tend to fluctuate between turn, random coil and α-helical conformations during the simulations of LL-37_MS_, LL-37_MT1_ and LL-37_MT2_.^38^ The differences in the LL-37's α-helicity observed in the simulations, and 3D-NMR experiments are most likely due to differences in the chemical structures of the molecules used to form the micelles. The negatively charged headgroups of SDS and D8PG, and their relatively shorter hydrophobic tails, compared to GMO, could potentially affect their self-assembly behavior and result in different interactions with LL-37. Additionally, electrostatic interactions among the negatively charged headgroups of SDS and D8PG and the positively charged amino acids of LL-37 could potentially stabilize a higher content of its α-helical secondary structure.

In good agreement with experimental observations, Fig. 4 also shows that the simulated C-terminal of LL-37 is unstructured.^38,39^ Analysis of the RMSD of the peptide's alpha carbon in the backbone as a function of the simulation time is shown in Fig. S6.† LL-37_rand_ shows the more substantial initial change in the RMSD because the peptide was obtained from a high-temperature simulation that denatured it. When quenched to room temperature, the number of conformations accessible decreased, leading to a reorganization of the peptide. LL-37_rand_ is also unstructured and will, therefore, have more conformational freedom compared to the other α-helical LL-37 simulated. Nonetheless, after an initial fast increase, the RMSD of LL-37_rand_ oscillates between 1 and 1.3 nm and appears to stabilize. The RMSD of LL-37_α_, LL-37_MT2_, and LL-37_MS_, however, display sharper variations along the RMSD, implying that the conformations are changing during the simulations. Sudden changes larger than 0.4 nm along the simulations are observed in Fig. S6a, d and e.† Analysis of peptides' snapshots during the simulations (Fig. S7†) in the proximity of the discontinuities in the RMSD highlight that they are mostly due to diffusion of the C-terminal. In the cases mentioned above, the position of the C-terminal before a substantial change in the RMSD is far from its position at the beginning of the simulation (Fig. S7†). Fig. S7† shows that when the RMSD is close to zero, the C-terminal aligns with the initial conformation while when the RMSD increases, it moves further away. Analysis of the RMSD after removing the alpha carbons of the C-terminal displayed in Fig. S8† demonstrates that the conformation of the other parts of the peptide, instead, remain mostly constant. The only exception is LL-37_rand_ that displays larger oscillations at the end of the simulation. These occur concurrently with the formation of α-helical and beta-turn secondary structures between 110 and 170 ns, as shown in Fig. 4.

As shown in the section above, the C-terminal part of the peptide is the part that displays the largest distance from the GMO/LL-37 micelles COM, which in combination with its lower α-helicity explains its higher mobility and confirms the experimental results.^38,39^ This section of the peptide contains four polar residues, two uncharged (THR and SER) and two charged (ARG and GLU), and two hydrophobic residues (PRO and VAL). The two hydrophobic amino acids have smaller hydrophobic side groups than isoleucine, leucine, and phenylalanine, and will therefore not have the same attachment strength to the GMO hydrophobic chains. Additionally, the four polar amino acids are all found at the end of the C-terminal. As hydrophobic moieties do not sandwich them, the C-terminal remains dynamic and can explore a larger conformational space.

While the 3D-NMR experiments on SDS and D8PG micelles and the here presented simulations (for LL-37_MS_, LL-37_MT2_, LL-37_rand_, and LL-37_α_) agree that the peptide bends between residues 13 and 15, the current simulations show that this bend can unwind the α-helical structure.^38^ Comparing the structures proposed by Wang and the present simulations (Fig. S9†), it appears that in LL-37_MT2_ and LL-37_MS_ the peptides' bend is qualitatively more pronounced on GMO than on SDS or D8PG micelles, probably due to a combination of the difference in the size of the micelles and the chemical structures of the molecules composing the micelles.^38^ In the simulations, this conformational change unfolded the α-helical secondary structure of the peptide between residue 13 and 15 (ILE-13, GLY-14, and LYS-15) in LL-37_MS_ and GLY-14 in LL-37_MT2_, decreasing the total α-helicity in LL-37 (Fig. 4b and c).

The peptide bend was observed in the same region also in LL-37_α_ and LL-37_rand_, suggesting that in this region, the peptide is flexible, allowing it to accommodate surfaces with different geometries. Therefore, the specific conformation of the polymer in this region will depend both on the interactions with other solutes or assemblies and on their structure. Generally, among these amino acids, glycine is known to reside in regions of proteins or enzymes that require conformational freedom to be able to perform their function.^79–81^ Therefore, the bend might have a role during the binding to bacterial surfaces or may be useful in other biological functions.

### LL-37 conformational entropy change upon binding to GMO

Table 1 presents the conformational entropy calculated for the LL-37 in water and their difference to that of LL-37 embedded in the GMO/LL-37 micelles. The conformational entropy was calculated by using the Schlitter's formula, which has been extensively used in previous works investigating peptides.^41,82–84^ The presented averages and standard deviations were obtained by splitting the 200 ns trajectory into 20 different sections of 10 ns (5000 frames) and then calculating the conformational entropy for each of them. A comparison of the values shows that LL-37_rand_ displays the highest conformational entropy, followed by LL-37_α_, LL-37_MS_, LL-37_MT1_, and LL-37_MT2_, respectively. This is sensible, as the LL-37 molecules in solution (LL-37_rand_ and LL-37_α_) are not constrained by interactions with the GMO molecules that restrain the internal degrees of freedom of the molecule. The largest conformational entropy change was observed for the transition between LL-37_rand_ and LL-37_α_, 385 kJ mol^−1^ (at 310 K). This change is much larger than the difference calculated between LL-37_α_ and the LL-37 interacting with GMO molecules (LL-37_MS_, LL-37_MT1_, and LL-37_MT2_) that range between 6 and 85 kJ mol^−1^ (Table 1). Nonetheless, these entropic changes can have a significant impact on the thermodynamics of the peptide as they lie between 2 and 33 times the thermal energy available at 310 K (∼2.58 kJ mol^−1^). The difference between these two values can be attributed to the changes in the α-helicity of the LL-37 upon self-assembly with GMO, discussed in the previous section. LL-37_MT2_, the most α-helical one (Fig. 4b) displays the lowest conformational entropy while LL-37_MS_ has the lowest α-helicity and the highest conformational entropy of the bound peptides (Fig. 4c). As LL-37_MT1_ and LL-37_MT2_ have lower conformational entropy than LL-37_MS_, another explanation for the change in conformational entropy could be associated with intermolecular interactions between the peptides (LL-37_MT1_ and LL-37_MT2_) when they are bound to the same micelle. These intermolecular interactions could restrict the internal degree of freedom of LL-37, thereby decreasing its conformational entropy.

**Table 1:** Conformational entropy of the different LL-37 simulated during this study calculated using the Schlitter's formula.^71^ In column 2 and 3, the differences in energy at 310 K (*T*Δ*S*, where *T* is temperature and *S* is entropy) using either LL-37_rand_ or LL-37_α_ as a reference state are presented

Simulation	Average (J K^−1^ mol^−1^)	Difference from LL-37_rand_ at 310 K (kJ mol^−1^)	Difference from LL-37_α_ at 310 K (kJ mol^−1^)
LL-37_rand_	9012 (±330)	0	385^a^
LL-37_α_	7771 (±273)	−385^a^	0
LL-37_MS_	7753 (±227)	−390^a^	−6
LL-37_MT1_	7735 (±210)	−396^a^	−11
LL-37_MT2_	7498 (±205)	−469^a^	−85^a^

a Significant result in comparison to reference state (0 point in the column), with *p* < 0.05.

The overall change in conformational entropy between LL-37_rand_ and the adsorbed LL-37 molecules is comparable to the values previously calculated for the adsorption of LL-37 onto a gold nanoparticle.^41^ Unfortunately, the change in the secondary structure of the LL-37 upon binding on the gold surface was not assessed, and thus, a direct comparison is not possible.

### Structural analysis of the GMO/LL-37 micelles

**Fig. 5:**
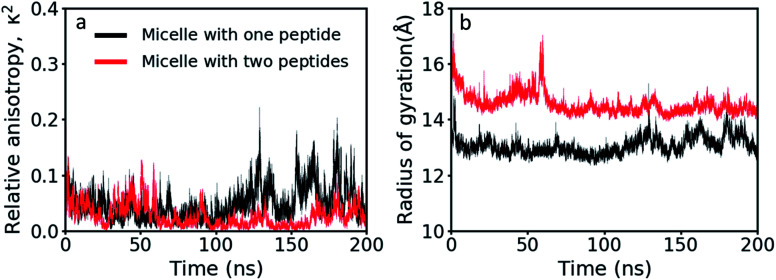
(a) Relative anisotropy values and (b) radius of gyration as a function of simulation time of the GMO/LL-37 micelles. In the images P1 is shown in red and P2 is in black.

Using the gyration tensor, the radius of gyration and the relative anisotropy factor were calculated for the two GMO/LL-37 micelles extracted from the large-scale simulation and are presented as a function of simulation time (Fig. 5). Of the two micelles, one contained a single LL-37 and 15 GMO molecules (P1), while the other contained two LL-37 and 14 GMO molecules (P2). Relative anisotropies oscillated between a minimum of 0.0 to a maximum of ∼0.2 (Fig. 5a). On average, the anisotropy factor was below 0.05 (0.047 for P1 and 0.025 for P2), indicating that the micelles are nearly spherical,^63^ in agreement with the experimental findings on this system.^45^ P1, however, displays larger oscillations in the anisotropy factor than P2, suggesting that it has higher conformational freedom. As the number of LL-37 molecules is double in the P2 micelles while the number of GMO molecules in P1 and P2 are similar, the reason behind the difference in these oscillations may be assigned to increased rigidity of the GMO-LL-37 micelle with a higher amount of LL-37 present. This correlates well with the conformational entropy measurements described above. The LL-37 interacting with P2 displayed a lower conformational entropy, which means that it lost more internal degrees of freedom than the LL-37 adsorbed onto P1, suggesting that the presence of two LL-37 increases the stability of the complex. The number of adsorbed LL-37 molecules also influences the radius of gyration, increasing it from 12.9 Å for P1 to 14.5 Å for P2 (Fig. 5b).

**Fig. 6:**
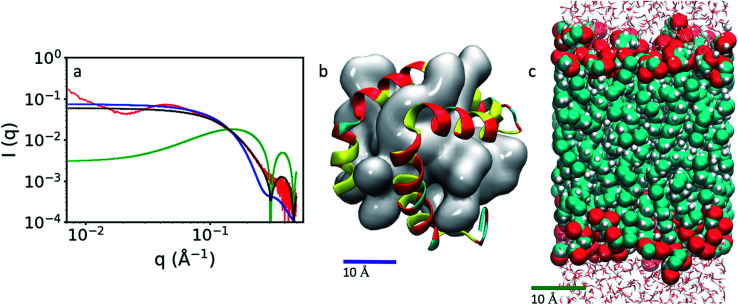
(a) SAXS profiles obtained experimentally (red curve, adapted from Mahsa *et al.*)^45^ and from the MD simulations where the blue line was obtained from the GMO/LL-37 micelles, the green line from the GMO bilayer and the black line by combining the spectra of the GMO/LL-37 micelles and the bilayer. The experimental curve was obtained from a solution containing 0.5 wt% GMO and 0.5 wt% LL-37 in water. (b) GMO/LL-37 micelle obtained from the MD simulations. In this image, the GMO molecules are shown as a continuous glossy surface and LL-37 is drawn using a cartoon representation. (c) GMO bilayer shown using a vdW representation: the carbon is colored in turquois, the oxygen in red and the hydrogen in white.

The SAXS profile calculated from the MD simulations for the GMO/LL-37 micelles and the one obtained experimentally at a 1/1 ratio of GMO and LL-37, shown in Fig. 6a, display significant differences.^45^ Below *q* of 0.1 Å^−1^ the simulated *I*(*q*) does not display any correlation with the experimental data because the size of the simulated systems is smaller than 50 Å, as shown in Fig. 6b and c. Instead, in this region, the constant overall scattering from the micelles was observed in the simulated *I*(*q*) curve, while the experimental curve has a hump around *q* ∼ 0.04 Å^−1^, a minimum at *q* ∼ 0.02 Å^−1^ and then increases again at lower *q*. From the analysis of the experimental SAXS data, complemented with cryogenic electron microscopy and dynamic light scattering studies, the presence of vesicles of various shapes potentially coexisting with small micelles was reported.^45^ Therefore it is not surprising that the simulated *I*(*q*) of the GMO/LL-37 micelles from the MD simulations does not follow the experimental *I*(*q*) of this system. Combining 21% of a SAXS *I*(*q*) profile calculated from the MD simulation of a hypothetical GMO bilayer (Fig. 6c) to 79% of the *I*(*q*) from MD simulation of the micelles leads to a curve that agrees with experimental data at *q* > 0.09 Å^−1^ (Fig. 6a) and reduces the RMSD, as shown in Fig. S10.† Overall, it appears that the system contains GMO/LL-37 vesicles and GMO/LL-37 micelles, as previously suggested.^45^

## Discussion

The MD simulations demonstrate the interactions between the amphiphilic lipid GMO with the antimicrobial peptide LL-37 at the molecular level. They show the formation of direct, water-continuous GMO/LL-37 self-assemblies. The hydrophobic amino acids of LL-37, mainly isoleucine, phenylalanine, and leucine, were mostly interacting with the aliphatic tail of the GMO molecules. Hydrogen bonding interactions between the hydroxyl groups present in the glycerol heads of GMO and the polar/charged amino acids of LL-37 were also observed. However, the polar groups preferentially interacted with the water molecules. LL-37 appeared to adopt this amphipathic conformation on micelles irrespective of the amphiphilic molecule chemistry and self-assembled structure, as similar conformations were reported for LL-37 self-assemblies with DPC, D8PG, and SDS.^38,39^

The percentage of α-helical secondary structure in LL-37 in the GMO/LL-37 micelles was lower in the current simulations than in the previous experimental NMR results using SDS, D8PC, and DPC. The difference was located in two specific regions: between residue 14 and 16 and between residue 29 and 31. As these two regions contain a positively charged amino acid (LYS-15 and ARG-29, respectively) and GMO does not carry a negative charge on its head group, in contrast to SDS, D8PG, and DPC, the electrostatic interactions among the positively charged residues present along the LL-37 backbone and the amphiphilic molecules head groups will be lower on GMO than on DPC, D8PG, and SDS. The positively charged LYS-15 and ARG-29 will be more likely to be constrained by interactions with negatively charged groups that could potentially force them into an α-helical conformation. Thereby, the chemistry of the head groups of the molecules composing micelles or vesicles can affect the secondary structure of the peptide and potentially its biological function.

As LL-37 is positively charged, the total charge of the resulting micelles will increase with its LL-37 content, increasing the electrostatic interactions with a negatively charged bacteria membrane.^16,26^ Furthermore, these self-assemblies would increase the local peptide load delivered to the bacterial membrane, which supports the proposed shuttle mechanism used to explain the higher antimicrobial activity of the self-assemblies compared to the individual peptides.^16,26^ The MD simulations also highlight that LL-37 bound in the GMO/LL-37 micelles displays a conformation that might facilitate adsorption onto the bacterial cell membrane. Eight out of the sixteen charged amino acids are located at a larger distance from the COM of the micelles than the average. Six of these amino acids are positively charged and can thus facilitate interactions with the negatively charged bacterial membrane.

The results presented on the LL-37 conformational entropy, show that its value is up to 82 kJ mol^−1^ lower when bound to a micelle than when in solution (at 310 K). For a molecule to interact with a surface, it needs to penetrate an environment with surface-structured water, impinging the internal degrees of freedom of the peptide. Atomic force measurements and simulations demonstrated that water forms structured layers on phospholipids membranes.^85,86^ According to previous simulations where the enthalpic and entropic contributions of a molecule adsorbing onto a mineral surface were separated, entering these water layers results in an enthalpic and entropic penalty.^87^ The former arises from the unfavorable removal of water molecules from the organized layers, while the latter is associated with a loss of flexibility and degrees of freedom of the molecule as it enters this constrained environment.^87^ Combination of these two changes leads to a transition energy barrier that will hinder adsorption. In this context, the conformational entropy loss associated with the adsorption of LL-37 to a bacterial membrane will be lower when it is bound to GMO than when in solution, a factor that could facilitate the adsorption of LL-37 onto bacterial membranes. This should be investigated in more detail in future work by using advanced sampling methods to obtain the free energy changes between adsorption of a free LL-37 and a GMO/LL-37 micelle on a model bacterial membrane.

## Conclusions

The MD simulations on GMOs and LL-37 at a weight ratio of 1/1 in water demonstrate their self-assembly into micelles. The formation of the micelles is driven by hydrophobic contacts amongst the aliphatic chains of GMOs and the hydrophobic amino acids in the LL-37 backbone. Phenylalanine, isoleucine, and leucine amino acids were found to dominate the interactions with the hydrophobic tail of the GMO molecules. Additionally, the charged and polar amino acids were found to interact with the glycerol head groups or remain in solution, free to interact with other molecules in solution or at the bacterial membrane.

Availability of the peptide's charged amine and guanidium groups from multiple LL-37 on the GMO/LL-37 micelles suggests that these self-assembled structures might facilitate interactions with bacterial membranes. Further, the loss in the conformational entropy of LL-37 upon binding to GMO, combined with the higher positive charge when multiple LL-37 are present on the GMO/LL-37 micelles, could decrease the transition energy associated with adsorption to surfaces, a process that should be explored in more depth in future work.

The presented results provide detailed insights into the self-assembly of GMO and LL-37 that leads to the formation of peptide nanocarriers and the effect of the interactions on the conformation of LL-37. The changes in the conformation of LL-37 and its conformational entropy suggest that these structures may facilitate its interactions with bacterial surfaces. The observations may drive the fine-tuning of self-assembled peptide nanocarriers to maximize their antimicrobial efficiency.

## Conflicts of interest

The authors declare that there are no conflict of interest.

## Supplementary Material

RA-010-C9RA10037G-s001
