# Supplementary material for: Self-assembly of glycerol monooleate with the antimicrobial peptide LL-37: a molecular dynamics study
Source: RSC Adv. 2020 Feb 26;10(14):8291–302. doi: 10.1039/c9ra10037g (PMC9049931; doi:10.1039/c9ra10037g)
Supplement: RA-010-C9RA10037G-s001 [file RA-010-C9RA10037G-s001.pdf]

Electronic Supplementary Material (ESI) for RSC Advances.
This journal is © The Royal Society of Chemistry 2020

Nano-self-assembly of glycerol monooleate with the antimicrobial peptide LL-37: A molecular dynamics study

R. Innocenti Malini[1*], M. Zabara[2], M. Gontsarik[2], K. Maniura-Weber[2], R. M. Rossi[1], F. Spano[1], S. Salentinig[2,3*]

[1] *Empa, Swiss Federal Laboratories for Materials Science and Technology, Laboratory for Biomimetic Materials and Textiles and* [2]*Laboratory for Biointerfaces Department Materials meet Life, Lerchenfeldstrasse 5, 9014 St. Gallen, Switzerland.*

[3]*Department of Chemistry, University of Fribourg, Chemin du Musée 9, 1700 Fribourg, Switzerland.*

* riccardo.innocentimalini@empa.ch
stefan.salentinig@unifr.ch

Supporting Information

Leucine (Leu) – Leucine (Leu) – Glycine (Gly) – Aspartic acid (Asp) – Phenylalanine (Phe) - Phenylalanine (Phe) – Arginine (Arg) – Lysine (Lys) – Serine (Ser) – Lysine (Lys) – Glutamic acid (Glu) – Lysine (Lys) – Isoleucine (Ile) – Glycine (Gly) – Lysine (Lys) – Glutamic acid (Glu) – Phenylalanine (Phe) – Lysine (Lys) - Arginine (Arg) – Isoleucine (Ile) – Valine (Val) – Glutamine (Gln) – Arginine (Arg) – Isoleucine (Ile) – Lysine (Lys) – Lysine (Lys) – Aspartic acid (Asp) – Phenylalanine (Phe) – Leucine (Leu) – Arginine (Arg) – Asparagine (Asn) – Leucine (Leu) – Valine (Val) – Proline (Pro) – Arginine (Arg) – Threonine (Thr) – Glutamic acid (Glu) – Serine (Ser)

Figure S1. Amino acid sequence composing LL-37.

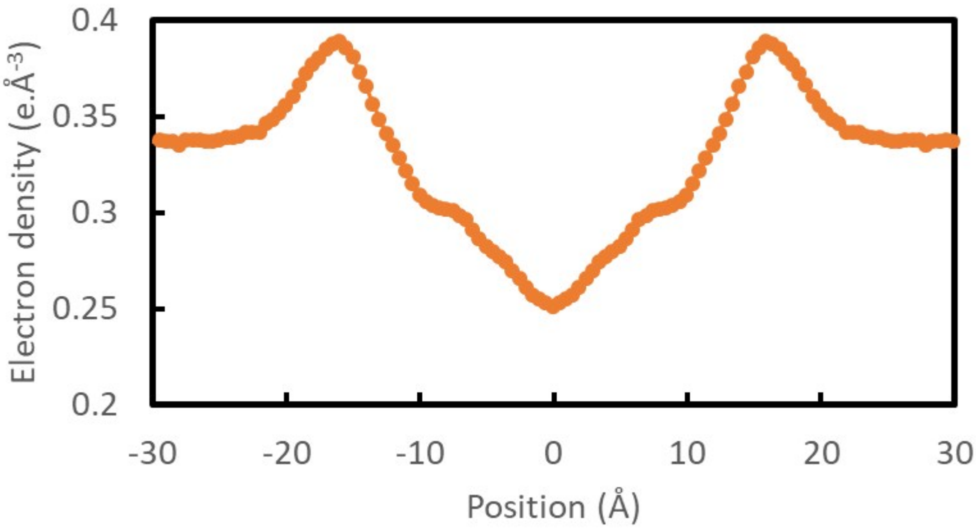

Figure S2. Average electron density of the system as a function of the position along the vector normal to the GMO bilayer surface.

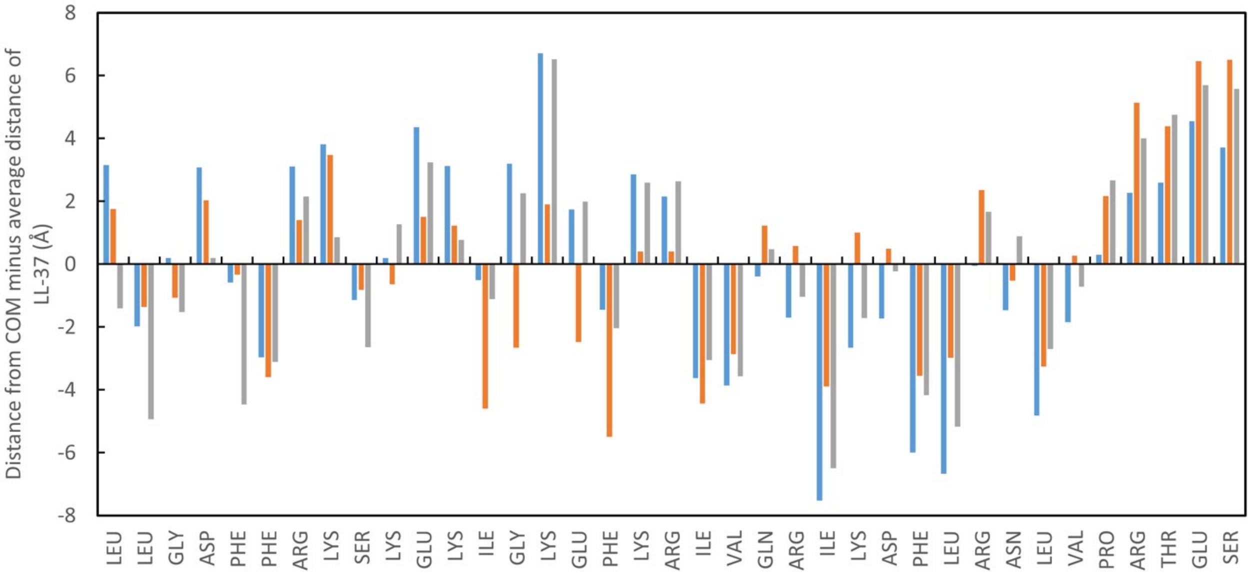

Figure S3. Distances of the center of mass of the different amino acids in LL-37 from the center of mass of the GMO/LL-37 micelles minus the average distance of the peptide.

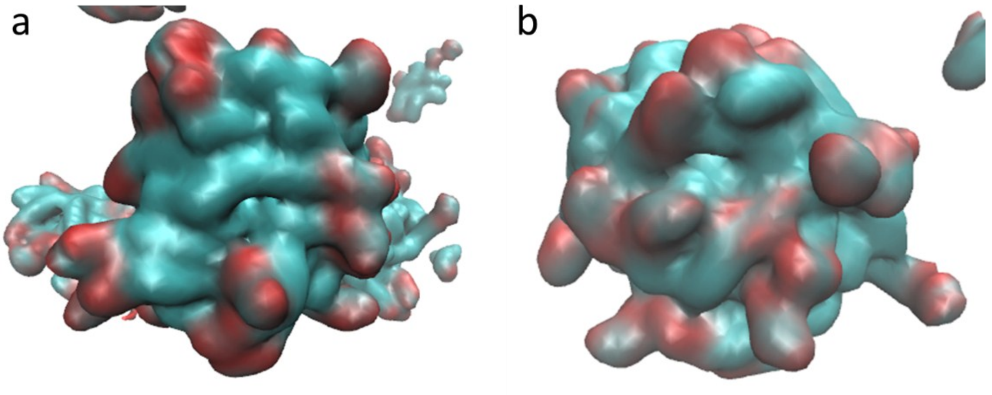

Figure S4. Surface images of the GMO aggregates where the carbon atoms are colored in cyan and the oxygen atoms in red. Hydrogen atoms present in the GMO and the water molecules are not shown for clarity. a) Hydrophobic region of the aggregate and b) hydrophilic one.

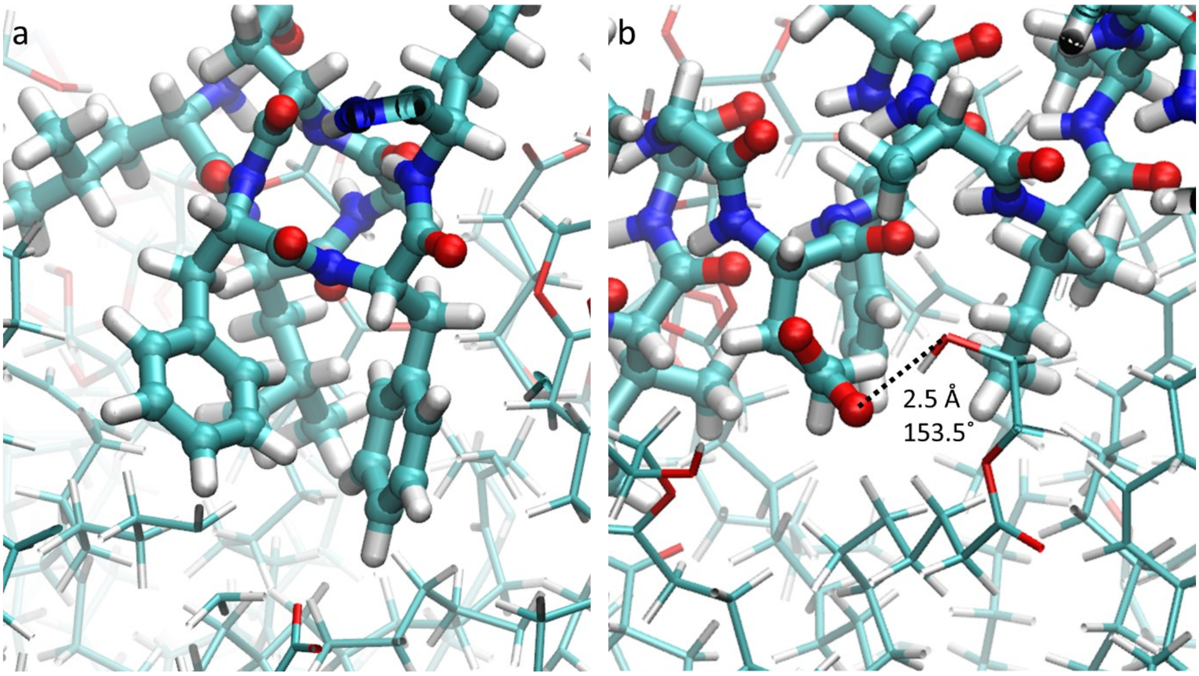

Figure S5. a) Example of hydrophobic contacts leading to the inclusion of the phenylalanine's cyclic ring into the aliphatic chains of the GMO molecules. b) Illustration of a hydrogen bond between a glutamic acid in the LL37 chain and the head group of a GMO molecule. In the image the water molecules are not shown for clarity. The peptide is displayed using a thick dynamic bond and van der Waals sphere representations while the GMO molecules are represented as thin dynamic bonds.

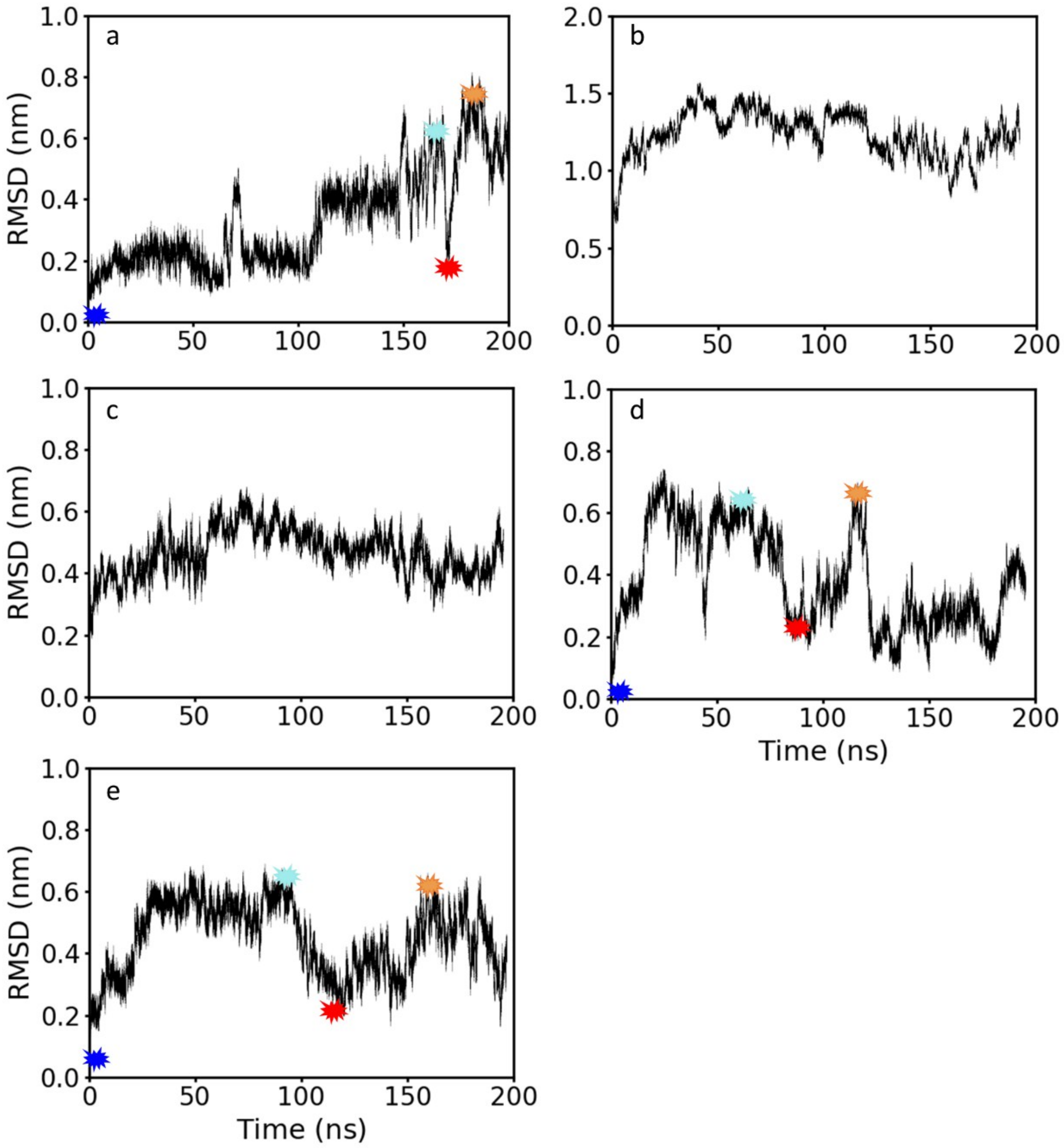

Figure S6. Root mean square deviation (RMSD) between the initial LL-37 structure and snapshots along the trajectory of a) a LL-37_{α} b) LL-37_{rand}, c) LL-37_{MT1} d) LL-37_{MT2} and e) LL-37_{MS}. The RMSD was calculated only using the α carbons in the backbone chain. The colored asterisks are time points where a snapshot of the trajectory was taken in order to understand the changes in the peptide configuration leading to the jumps in the RMSD value. These representations are shown in Figure S5.

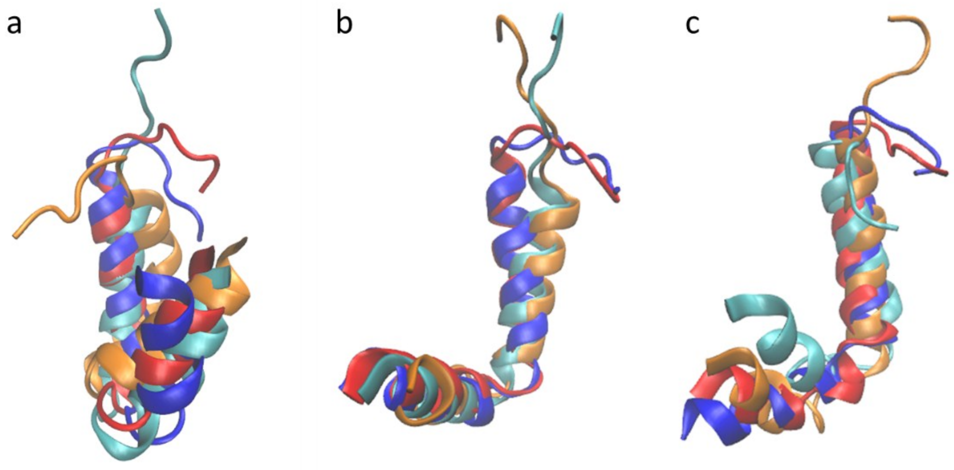

Figure S7. Snapshots of a) LL-37 at different time points during the simulation. a) was along the trajectory depicted in figure S4a, b) is from figure S4d and c) was obtained from figure S1e. It is clear from the images that the main changes in the peptide backbone during the simulations occur in the C-terminal, which is unstructured and free. The rest of the peptide remains stable throughout the simulation.

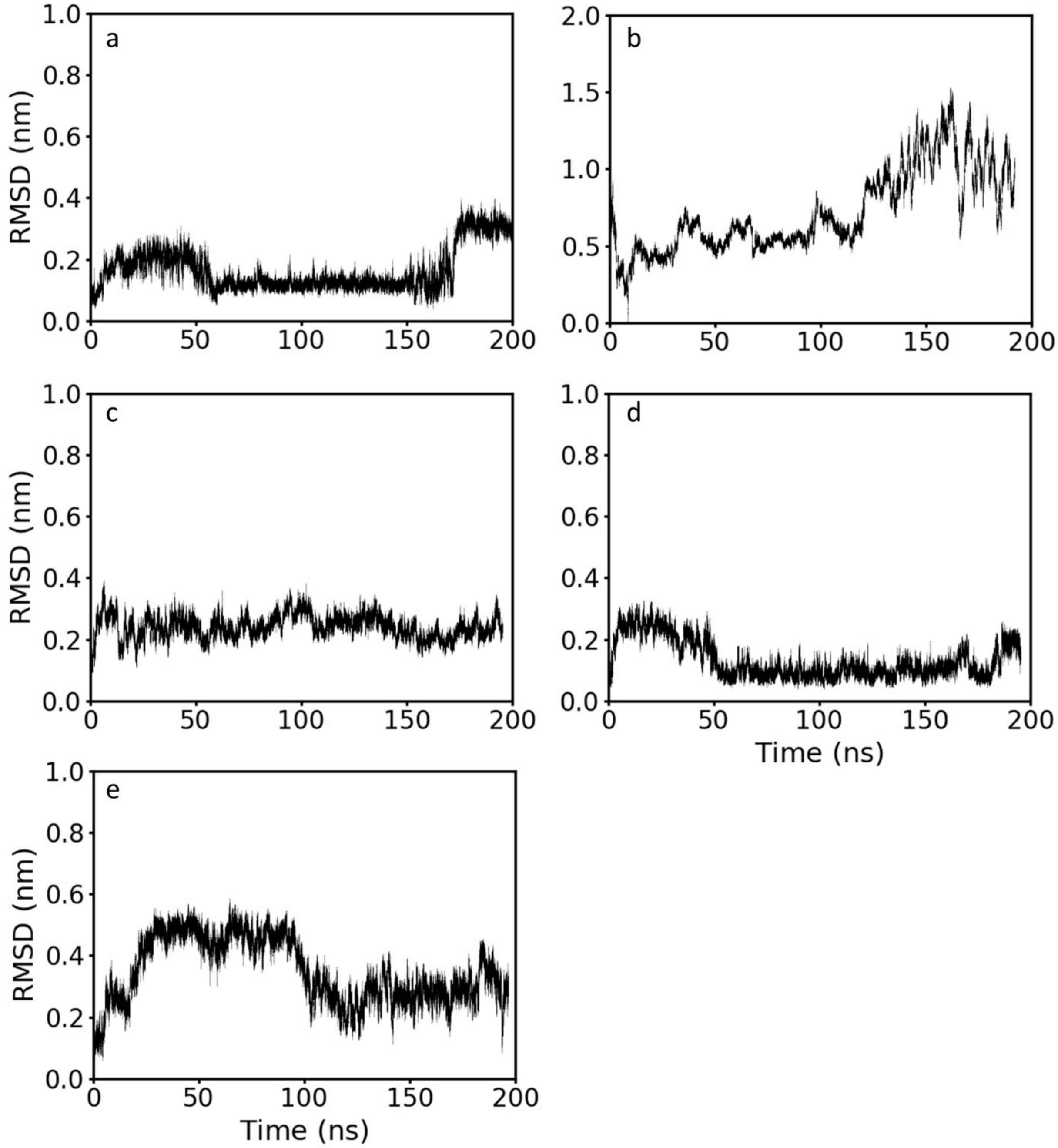

Figure S8. RMSD between the initial LL-37 structure and snapshots along the trajectory of a) a LL-37_{α} b) LL-37_{rand}, c) LL-37_{MT1} d) LL-37_{MT2} and e) LL-37_{MS}. The RMSD was calculated only using 28 α carbons in the backbone chain, discarding the ones in the C-terminal of the peptide.

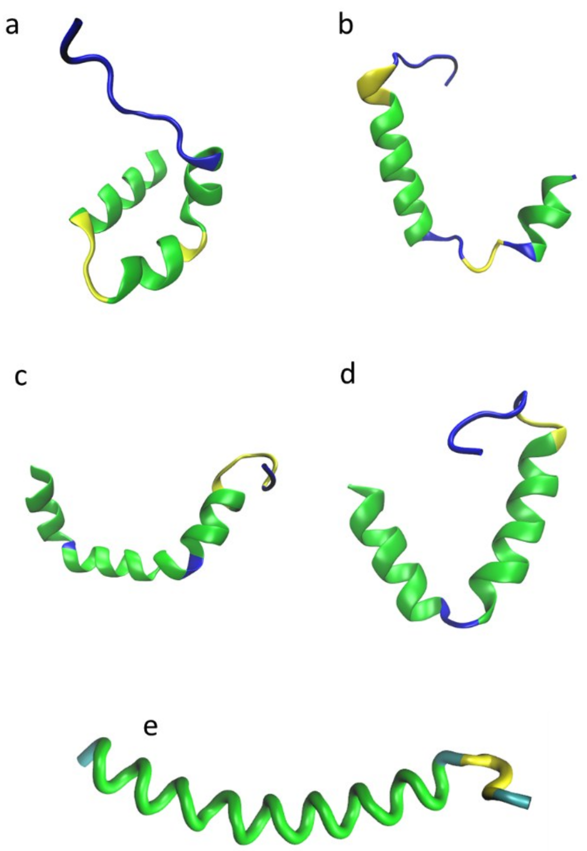

Figure S9. Snapshot of a) LL-37$_{\alpha}$, b) LL-37$_{MS}$, c) LL-37$_{MT1}$, d) LL-37$_{MT2}$, and e) LL-37 proposed by Wang et al on SDS and D8PG micelles.[1]

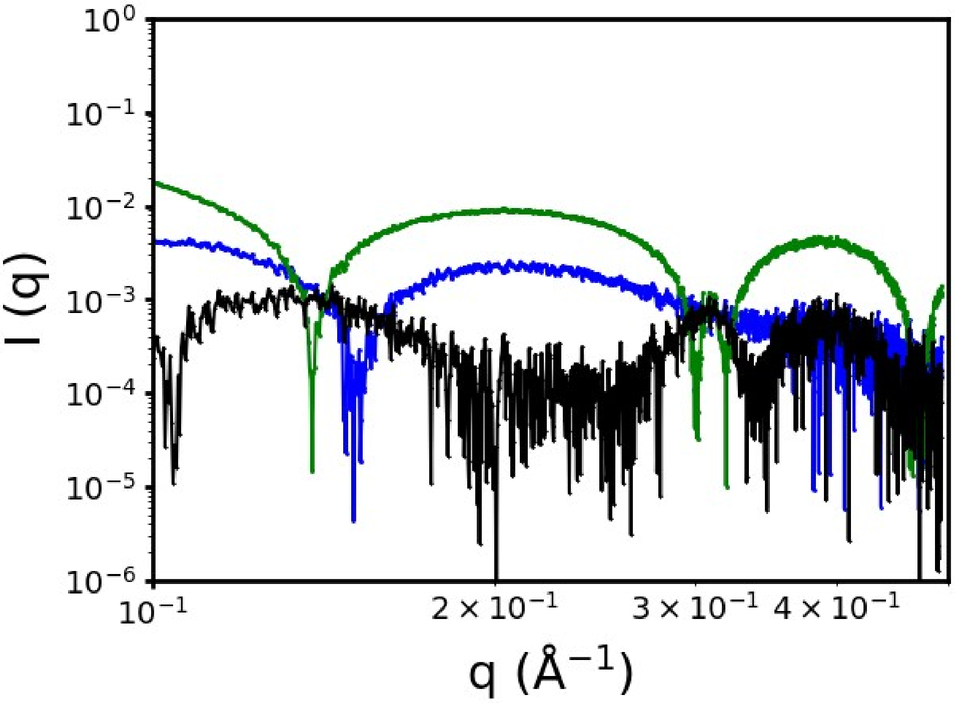

Figure S10. RMSD of the simulated SAXS profiles compared to the one obtained experimentally. Blue is the RMSD obtained for the micelles, green for the bilayer and black for their combination.
